# Efficacy of Nationwide Curfew to Encounter Spread of COVID-19: A Case From Jordan

**DOI:** 10.3389/fpubh.2020.00394

**Published:** 2020-08-21

**Authors:** Moawiah Khatatbeh

**Affiliations:** Department of Basic Medical Sciences, Faculty of Medicine, Yarmouk University, Irbid, Jordan

**Keywords:** COVID-19, curfew, Jordan, lockdown, Middle East

## Introduction

As of 20th June 2020, more than 8.6 million people are confirmed to have the novel COVID-19 in more than 200 countries (1). To stop the spread of COVID-19, countries have responded in varied ways. However, the response depended on several factors, of which, the number of cases and timing of interventions are the most crucial.

In Jordan, the first case of COVID-19 was discovered on 2nd March 2020 (2). The government started immediate interventions. Patients were immediately and completely isolated in specialized hospitals and quarantine was initiated as per official guideline to the medical professionals and using all personal protective equipment (3).

It has been reported in the literature that individuals with other co-morbidities are at a greater risk for catching COVID-19 infection (4). The prevalence of chronic non-communicable diseases in Jordan is considered high. For example, 38% of deaths is attributed to cardiovascular diseases (5). In addition, a recent study from Jordan revealed that the prevalence of diabetes in men and women aged ≥25 years was 32.4 and 18.1%, respectively (6). Therefore, the government started using all media channels including social media, TV, press advertisements, and smart mobile applications to raise the attention of people about the disease, its mode of transmission, and preventive measures.

## Jordan's Response to the Increased Number of COVID-19 Cases

During the first 2 weeks, Jordan had only one confirmed case of COVID-19 before starting to diagnose new cases in the third week (from 14 to 20 March 2020), after which the country started implementing vital interventions to combat the spread of the disease. These interventions were empowered by the activation of the National Defense Law on 17th March 2020. This law stipulates that, “upon a decision and a Royal Decree, a National Defense Law shall be passed in case of emergency that would threaten the national security or public safety in all parts of the Kingdom or in a region due to war, disturbances, armed internal strife, public disasters or the spread of a pest or epidemic” (7). The activation of the law led to suspension of the studies at educational institutions, closure of borders, stopping prayers in places of worship, and all large gatherings were banned.

After 19 days of discovery of the 1st case in Jordan (21 March 2020), 15 new cases were confirmed heading a total of 98 cases in the country, which constituted a red flag sign. On that day, several additional interventions have been taken, of which implementing complete nationwide curfew (24/24 h) for 3 days−22–24 March—was of great efficiency (8). After these 3 days of nationwide lockdown (from 25 March until the moment of writing this report), the government has implemented several days of complete curfews over weekends, in addition to the daily partial lockdowns.

During the curfew time, no one was allowed to move except the medical and nursing staff, police, and the armed forces. The government announced that these nationwide curfews will enable epidemiological investigation teams to trace patients' contacts and test them. Moreover, the complete curfew promotes social distancing and minimizes the number of new infections.

## Impact of Curfew Measures

As of 20th June 2020, the total number of cases of COVID-19 in the country was 1,008 (2). This denotes that the curfew measures were effective and efficient. However, to assess the effectiveness of the lockdown, a comparison between Jordan and other countries in the Middle East would be beneficial in terms of the total number of cases relative to date of confirming the first case and time of implementing lockdowns. The number of cases divided by the total number of population will be also considered to understand the magnitude of the disease in each country. The number of population for each country was figured from the world live population meter, and total number of cases was figured from Johns Hopkins University's Coronavirus Resource Center, both measures as of 20th June 2020.

Results in Table 1 show the latest statistics of COVID-19 in 12 Middle Eastern countries. It is noteworthy to say that countries including Syria and Palestine were excluded from this analysis because of their limited ability to contain the disease due to economic and political reasons.

**Table 1:** Number of cases of COVID-19 in 12 countries in the Middle East as of 20th June 2020.

**Country**	**Date of first confirmed case**	**Number of cases at curfew point**	Current total number of cases^*^	Number of cases/100,000 population^**^	**Ratio of number of cases/100,000 population countries vs Jordan**
Jordan	02 Mar 2020	98	1,008	11	-
Iran	19 Feb 2020	11,364	200,262	238	21.6
Turkey	11 Mar 2020	47,029	185,245	217	19.7
Saudi Arabia	02 Mar 2020	511	150,292	425	38.6
UAE	28 Jan 2020	611	44,145	437	39.7
Qatar	29 Feb 2020	562	85,462	3,015	274
Bahrain	16 Mar 2020	211	20,916	1,388	126.1
Kuwait	26 Feb 2020	176	38,678	782	71.1
Oman	25 Feb 2020	20	28,566	422	38.3
Lebanon	21 Feb 2020	368	1,510	19	1.7
Egypt	14 Feb 2020	363	52,211	51	4.6
Iraq	15 Mar 2020	124	27,368	64	5.8

* *Figured from https://coronavirus.jhu.edu/map.html as of 20/06/2020*.

** *Figured from https://countrymeters.info/en/World#population_clock as of 20/06/2020*.

## Discussion

Results in Table 1 show that Jordan has the lowest number of COVID-19 cases compared to other countries in the Middle East. Despite Jordan having the disease before some countries like Turkey and Bahrain and having the disease on the same day as Saudi Arabia, these countries had very high number of cases compared to Jordan.

The lowest ratio of cases to the number of population was seen in Jordan (11/100,000) compared to all other countries. Qatar, Bahrain, UAE, Saudi Arabia, Turkey, and Iran had 3,015, 1,388, 437, 425, 238, and 217/100,000 population, respectively. Interestingly, among all countries, Qatar and Bahrain had the largest number of cases proportionate to the number of population.

The crucial factors in minimizing the infection rate are numerous. These factors may include demographic characteristics, precautions taken, public commitment, firmness in implementing measures, public awareness of the disease, national vaccinations' programs, and many other factors. However, in case of COVID-19, timing of implementing the lockdown is immensely crucial. In Jordan, the implementation of the nationwide curfew was stated as one of the weapons used in the battle against COVID-19 (9, 10). The country has implemented strict nationwide curfew measures at early stages compared to other countries. This early start and the relative low number of cases enabled the epidemiological investigation teams to detect the primary and secondary contacts and test them. In fact, many of the detected cases in Jordan were from those contacts. It is worth noting that the diagnostic tests are available in Jordan as more than 400,000 tests were carried out until the moment of preparing this report (2).

It seems that this was crucial in decreasing the infection rate. The effectiveness of the curfew was enhanced by the closure of all entry borders including the airport and the compulsory 14-days quarantine for all individuals arriving to Jordan within 3 days preceding closing the borders. Similarly, Oman has initiated curfew when they had only 20 cases. However, they have now a huge number of cases compared to Jordan. This likely refers to less strictly adopted curfew measures. All other countries included in this analysis implemented curfew measures at later stages and when they had larger number of cases compared to Jordan, which may explain their current statistics.

Disease statistics from Jordan reveal that interventions implemented, and precautions taken, especially the strict nationwide curfew, were successful in preventing the spread of the disease (9, 10). Despite the high prevalence rates of chronic diseases in the Jordanian population that put these vulnerable groups at higher risk for catching COVID-19 infection, the preventive measures and precautions taken by the country were effective in decreasing its spread. Among all countries in the Middle East, Jordan is in principle better positioned to respond to the outbreak of COVID-19 (11, 12). The effectiveness of the interventions is manifested by having the lowest number of cases in the Middle East and the country started documenting only few cases daily after 3 weeks of initiating the curfew. Nevertheless, implementing strict and frequent nationwide lockdowns has its price of impacting the economy due to the increased unemployment rate and losses in the gross domestic product especially in a country with limited resources, like Jordan, despite the latest World Bank classification of Jordan as an upper middle-income country (13). These adverse effects of the lockdown may have an impact on public mental health as a recent review reported that subsyndromal mental health problems are a common response to COVID-19 pandemic (14). The good news is that Jordan started relieving lockdown measures due to the controlled and comfortable epidemiological situation.

## Author's Note

Covid-19 has invaded more than 200 countries around the world. Countries have responded in varied ways to combat its spread. Western and developed countries are extremely suffering from huge cases numbers. However, in the Middle East, Jordan has implemented strict measures in fighting against the disease. Initiating nationwide curfew in the country, parallel with other interventions, has been effective in decreasing infection rate in Jordan. This effectiveness is manifested by having the lowest number of cases among Middle Eastern countries. Such interventions are important to be viewed and recognized by other countries, specially the developing ones, to fight against such pandemics.

## Author Contributions

The author confirms being the sole contributor of this work and has approved it for publication.

## Conflict of Interest

The author declares that the research was conducted in the absence of any commercial or financial relationships that could be construed as a potential conflict of interest.
